# Internal Migration, Mealtime Social Disconnection, and Alcohol Use Are Linked to Poor Dietary Habits Among Peruvian Medical Students

**DOI:** 10.3390/healthcare14040433

**Published:** 2026-02-09

**Authors:** Alba Navarro-Flores, Josue Humpire-Belizario, Frances Condori, Kevin Pacheco-Barrios

**Affiliations:** 1 Institute of Psychiatric Phenomics and Genomics (IPPG), LMU University Hospital, LMU Munich, 80336 Munich, Germany; 2 International Max Planck Research School for Translational Psychiatry (IMPRS-TP), 80804 Munich, Germany; 3 Escuela de Medicina, Universidad Cesar Vallejo, Trujillo 13001, Peru; 4 IvyBridge Program, Philltec, Lima 15023, Peru; 5 Neuromodulation Center and Center for Clinical Research Learning, Spaulding Rehabilitation Hospital and Massachusetts General Hospital, Harvard Medical School, Boston, MA 02138, USA; 6 Unidad de Investigación para la Generación y Síntesis de Evidencias en Salud, Vicerrectorado de Investigación, Universidad San Ignacio de Loyola, Lima 15023, Peru

**Keywords:** dietary habits, medical students, internal migration, social isolation, eating alone, alcohol use, Peru

## Abstract

**Background**: Medical students are a vulnerable population with elevated cardiometabolic and mental health risks. Dietary habits are key modifiable behaviors that may mitigate these risks, yet social and contextual determinants such as internal migration, social disconnection, and alcohol use remain underexplored, particularly in middle-income settings. This study examined dietary habits among Peruvian medical students and evaluated the associations of internal migration, mealtime social disconnection, and alcohol consumption with poor habits. **Methods**: We conducted a cross-sectional study of 223 medical students in Peru. Dietary habits were assessed using a validated nutritional habits questionnaire adapted for the target population (Diet History Questionnaire test, Peruvian adaptation—DHQ-P). The score ranged from 0 to 58, with poor habits defined as <30 based on previous studies and the sample median. Mealtime social disconnection, internal migration, and alcohol use were explored as associated factors. Multivariable Poisson regression models with robust standard errors were used to estimate adjusted prevalence ratios (aPRs), controlling for age, gender, medical school semester, and income. Causal mediation analysis evaluated whether mealtime social disconnection mediated the association between internal migration and dietary habits. Sensitivity to unmeasured confounding was assessed using E-values. **Results**: Poor dietary habits affected approximately 47% of students. Internal migrants comprised 44% of the sample, 35.8% reported eating all meals alone, and 67% reported alcohol use more than once per month. Each additional meal eaten alone was associated with a higher prevalence of poor habits (aPR 1.17, 95% CI 1.02–1.38), and eating all meals alone also increased the prevalence (aPR 1.14, 95% CI 1.02–1.28). Similarly, recent internal migration (aPR 1.38, 95% CI 1.04–1.83), lifetime internal migration (aPR = 1.85; 95% CI, 1.16–2.95), and higher frequency of migration (aPR = 1.17; 95% CI, 1.01–2.38) were linked to poor dietary habits. Moreover, alcohol consumption (aPR 2.06, 95% CI 1.60–2.67) was independently associated with poor dietary habits. Mealtime social disconnection partially mediated 19% of the migration–dietary habits association (*p* = 0.03). Associations were robust to unmeasured confounding (E-values 1.34–2.23). **Conclusions**: Poor dietary habits are highly prevalent among Peruvian medical students and are independently associated with internal migration, mealtime social disconnection, and alcohol use. Addressing social eating contexts and migration-related vulnerabilities may offer novel opportunities to improve dietary behaviors among future physicians.

## 1. Introduction

Medical education represents a critical period of the future healthcare workforce, shaping clinical practice, health policy, and population health outcomes for decades to come. Despite their central role, medical students constitute a vulnerable population, consistently exposed to high academic demands, psychological stress, irregular schedules, and early professional socialization that sometimes expects unhealthy behaviors [1]. A growing body of evidence indicates that medical students exhibit elevated risks of cardiovascular risk factors [2], poor mental health [3,4], sleep disturbances [5], and burnout [6], which often begin during training and persist into professional life.

Dietary habits represent key modifiable behavioral factors that may buffer these vulnerabilities. Among medical students, poor dietary patterns characterized by low intake of fruits and vegetables, high ultra-processed food consumption, irregular meals, and frequent skipping are strongly linked to obesity, metabolic syndrome, depression, impaired cognitive performance, elevated inflammation, and suboptimal academic outcomes [7,8,9,10]. These patterns are particularly concerning, as physicians’ personal health behaviors influence their counseling credibility and practices with patients [11].

Dietary habits are key modifiable behavioral factors that may buffer many of these vulnerabilities. Healthy dietary patterns are strongly associated with improved cardiometabolic health, lower systemic inflammation, and better mental well-being [7], whereas poor dietary habits have been linked to obesity, metabolic syndrome, depression, and impaired cognitive performance [8,9]. Among medical students specifically, multiple studies from diverse settings report suboptimal dietary habits, characterized by low consumption of fruits and vegetables, high intake of ultra-processed foods, irregular meal patterns, and frequent meal skipping [10]. These patterns concern not only the current health of students, but also because personal health behaviors in physicians influence their counseling practices and credibility with patients [11].

Previous research has identified several contributors to unhealthy lifestyle behaviors among medical students, including heavy academic workload, high perceived stress, night shifts, and irregular class schedules, which disrupt circadian rhythms and meal timing [12]. However, important social and contextual determinants remain underexplored. In particular, internal migration, social isolation, and alcohol use have received limited systematic attention in the literature regarding medical student health risks.

Internal migration for educational purposes is increasingly common worldwide, particularly in countries with centralized medical education systems. Migration can disrupt established food environments, family support networks, and cultural eating practices, potentially increasing reliance on convenience foods and solitary eating [13,14]. Yet, few studies have examined how internal migration relates to dietary habits among medical students. Similarly, social isolation, operationalized through reduced shared meals or eating alone, has been associated with poorer dietary intake and adverse health outcomes in general populations [15,16,17], but has rarely been evaluated as a determinant of dietary habits in medical trainees. Alcohol consumption, which is prevalent among medical students and often co-occurs with stress and social disruption [18,19], has also been linked to unhealthy dietary patterns and meal skipping [20], though its independent contribution to dietary habits remains insufficiently characterized in this population.

These gaps are particularly evident in middle-income countries, where empirical data on medical students’ dietary habits and their social determinants remain scarce. This limitation is notable in countries such as Peru, characterized by highly centralized medical education [21], substantial internal migration, and remarkable geographic and gastronomic diversity [22]. Internal migration to urban academic centers may substantially alter food access, eating routines, and social contexts, yet these dynamics have not been systematically studied in medical students. Understanding dietary patterns and identifying modifiable behavioral and social risk factors, such as mealtime social disconnection and alcohol use, is essential for informing preventive strategies within medical schools and teaching hospitals, with potential long-term benefits for physician health and patient care.

Guided by prior literature and contextual considerations, we conceptualized internal migration as a multidimensional exposure encompassing recent educational migration and cumulative migration history. Based on our conceptual framework (Supplementary Figure S1), internal migration may be associated with diet quality both directly and indirectly through disruptions in daily routines and living conditions. In particular, migration may alter meal companionship patterns (e.g., increased frequency of eating alone), reshape the food environment (e.g., limited access to familiar or home-prepared foods), and increase time constraints related to academic demands and adaptation to new settings. These factors may, in turn, influence dietary habits and contribute to poorer diet quality. Based on this framework, we further considered alcohol use as a behavioral mediator that may arise in the context of disrupted routines and social environments and negatively affect dietary behaviors. Age, gender, income, medical school semester, and university were treated as potential confounders of these relationships.

Therefore, the aims of this study were to describe the dietary habits among Peruvian medical students and to examine the associations of internal migration, mealtime social disconnection, and alcohol consumption with poor dietary habits. We formulated the following hypotheses: (1) Medical students who have recently migrated for educational purposes or who have a greater cumulative migration history would exhibit poorer diet quality compared with non-migrant peers. (2) Migrant students would report a higher frequency of meals eaten alone. (3) Eating alone would be associated with poorer diet quality and partially explain observed differences between migrant and non-migrant students. Medical students represent a particularly relevant population in which to examine these hypotheses, not only because of the high academic demands of training, but also because dietary habits established during medical education may persist into professional life. As future physicians, medical students play a critical role in health promotion and serve as role models for patients, and medical schools represent structured institutional settings in which targeted, scalable interventions, such as protected meal times, shared dining spaces, or nutrition-focused programs, could be implemented to promote healthier dietary behaviors.

## 2. Materials and Methods

### 2.1. Study Design and Sample Selection

This study employed an observational cross-sectional design based on an online survey administered to medical students from nine universities in Peru. In Peru, undergraduate medical education spans seven years (14 semesters), comprising three years of pre-clinical training (1st–6th semesters), three years of clinical training (7th–12th semesters), and a final year of supervised clinical practice (internship) corresponding to the 13th and 14th semesters.

Eligible participants were medical students aged 18 years or older enrolled between the 1st and 12th semesters at the time of the study. Students in the internship year were excluded due to the distinct characteristics of their curriculum, including structured hospital-based schedules and access to institutional meals, which could introduce systematic differences in dietary patterns and limit comparability with non-internship students.

Data were collected between July and September 2024 using an online questionnaire developed and administered via Google Forms. Participants were recruited through a snowball sampling strategy, initiated by direct contact at university campuses, dissemination through student social media groups, and peer-to-peer referrals. No financial or non-financial incentives were offered for participation.

The online survey consisted of four sequential sections: (1) a brief description of the study objectives and procedures; (2) a digital informed consent form; (3) sociodemographic questions, including age, gender, academic semester, income, and migration status; and (4) a validated questionnaire assessing dietary habits. Access to the survey was conditional upon providing informed consent, which was obtained electronically from all participants prior to data collection.

The study protocol was reviewed and approved by the Ethics Committee of César Vallejo University, Trujillo, Peru. All procedures were conducted in accordance with national and international ethical standards for research involving human participants.

#### 2.1.1. Sampling and Bias

At each participating campus, we used a convenience-based seed selection strategy, inviting initial ‘seeds’ among students who were present in common areas (e.g., libraries, cafeterias, classrooms) and who were part of social media groups of medical students (e.g., university-based scientific societies) who met the basic inclusion criteria of being currently enrolled and aged ≥18 years. From these seeds, we asked each participant to invite peers from their personal networks (snowball sampling). Recruitment procedures were harmonized across sites, with the same inclusion criteria, survey link, and study information, although the absolute number of seeds per campus varied according to local enrollment size and feasibility. Because the survey was administered via an online platform, we implemented several measures to reduce duplicate responses. Each participant could access the questionnaire using the same device only once; we did not give incentives, either monetary or student credits, that might encourage multiple submissions, and we screened the raw data for implausible or duplicate patterns (e.g., identical time stamps, IP addresses, or near-identical response sets). Despite these measures, we acknowledge that our non-probabilistic snowball sampling and the potential for undetected duplicate entries may limit the representativeness of the sample and could introduce selection and information bias. These limitations are explicitly considered when interpreting our findings.

#### 2.1.2. Sample Size Calculation

The minimum required sample size was estimated assuming a finite population of medical students (N = 1025) enrolled at the index university. We used a 95% confidence level (Z = 1.96), a margin of error of 5% (E = 0.05), and an expected population proportion of 23%, based on prior national evidence reporting the prevalence of internal migration among Peruvian medical students. Using these parameters, the calculated minimum sample size was 217 participants. This target sample size was achieved and slightly exceeded in the final analytical sample (n = 223), ensuring adequate precision for the primary association analyses. The assumed population proportion was derived from the study by Chambergo-Michilot et al. [21], which examined internal migration for medical education across Peruvian medical schools.

### 2.2. Outcome

The study outcome was the dietary habit score calculated using a standardized questionnaire. The food frequency questionnaire applied was a modified version of the test version of the NCI Diet History Questionnaire (test-DHQ) [23]. This questionnaire has been adapted to the Peruvian dietary culture and food availability (DHQ-P), and has been previously used in local research [24,25,26]. It consists of 32 questions clustered into 10 domains. Internal consistency of the DHQ-P in the present sample was evaluated using Cronbach’s alpha. The scale demonstrated acceptable reliability (α = 0.74), supporting its use for group-level analyses in this population. The quality of the habits was either adequate or inadequate; each adequate item was rated with either 1 or 2 points (see Supplementary Methods S1 and S2). The maximum score of the test is 58 points. To address potential overlap between exposure and outcome definitions, we used a modified DHQ-P score that excluded all items related to “meal companionship.” Poor diet quality was defined as a modified DHQ-P score < 30. This cut-off has been used in previous studies employing the DHQ-P [24,25,26] and corresponds approximately to the median of the score distribution. In our sample, the DHQ-P score showed a near-symmetrical distribution with a median close to 30, supporting the use of this threshold to distinguish relatively poorer from better dietary habits and facilitate comparability across previous studies.

### 2.3. Exposures

**Internal migration**: Lifetime internal migration was defined as having moved from the city of birth at least once in their lifetime. Because internal migration is common in Peru [21], we additionally asked whether moving was due to the pursuit of their medical studies, to ensure that the migration was recent, as reported in previous studies [21,27,28]. This variable represented a more “recent internal migration” and was the main internal migration outcome analyzed in this study. Furthermore, we asked the participants to name the number of cities in Peru where they had lived and used this information to construct a variable named “frequency of migration”, defined as the number of times a participant had moved to a different city.

**Mealtime social disconnection**: Questions regarding company during main meals (breakfast, lunch, and dinner) were used. Mealtime social disconnection was defined as reporting to eat alone in one of the meals, with three levels of severity according to the number of meals. A dichotomous variable was created for those who reported eating the three meals alone (all meals alone, yes/no). This variable was used in previous studies as a proxy of objective social isolation behavior [29] and reflects an objective measure of meal companionship patterns rather than subjective loneliness or perceived social isolation. Subjective loneliness was not assessed in this study.

**Alcohol use**: It was measured using a question about the frequency of alcohol use. The variable was classified as adequate when participants denied using alcohol or only drinking alcohol up to once per month. Using alcohol more than once a month was considered inadequate. A similar variable was used in previous studies as a very sensitive surrogate of unhealthy lifestyle [30].

### 2.4. Other Covariates

The relevant confounders included were age, self-reported gender (male/female), semester of the medical training, and weekly income. Weekly income was reported in the Peruvian currency “Sol”. One Peruvian Sol (symbol: S/, singular: sol, plural: soles) is equivalent to 0.30 United States Dollars (USD). Three categories were created based on the reported values: low weekly income, up to 150 soles (around 44.6 USD); medium, between S/150-500 (44.6–148.6 USD); and high, more than S/500 (>148.6 USD). The median weekly income was 150 soles, which is enough to consume three meals per day, so it was used as a cutoff to divide low vs. medium weekly income. The living wage considered sufficient for a family of four in the internal regions of Peru (outside Lima) is 2000 soles per week [31]. Therefore, we used 500 soles per week as the cut-off for medium vs. higher weekly income for a medical student.

### 2.5. Statistical Analysis

Descriptive statistics were used to characterize the study population overall and by migration status. Continuous variables were summarized using medians and interquartile ranges (Q1–Q3) due to non-normal distributions, and categorical variables using frequencies and percentages. Group comparisons were performed using the Wilcoxon rank-sum test for continuous variables and the Pearson chi-squared test for categorical variables.

The primary outcome was poor dietary habits, defined a priori as a score < 30 on a validated nutritional habits scale (DHQ-P, range 0–55) without considering the three “meal companionship” questions. Associations between poor dietary habits and the main exposures—mealtime social disconnection, internal migration, and alcohol consumption—were examined using Poisson regression models with robust standard errors. Mealtime social disconnection was modeled both as an ordinal exposure (number of meals eaten alone per day, range 0–3) to evaluate dose–response relationships and as a binary exposure (eating all meals alone vs. sharing at least one meal). Alcohol consumption was dichotomized as >1 time per month vs. ≤1 time per month, and internal migration was defined as migration for educational purposes.

Crude and multivariable-adjusted prevalence ratios (PRs) with 95% confidence intervals (CIs) were estimated. Covariates included in adjusted models—age, gender, medical school semester, and weekly income—were selected *a priori* based on theoretical relevance (Supplementary Figure S1) and prior literature rather than statistical significance testing. Model assumptions were evaluated by assessing the linearity of continuous covariates on the log scale, examining influential observations using standardized residuals and leverage diagnostics, and verifying the absence of model convergence issues, consistent with recommended practices for modified Poisson regression with robust variance estimation. Multicollinearity was assessed using variance inflation factors, with no evidence of problematic collinearity observed.

Effect modification was evaluated by including multiplicative interaction terms between internal migration and both mealtime social disconnection and alcohol consumption. Interaction effects were assessed using likelihood ratio tests, comparing nested models with and without interaction terms.

To investigate potential mechanisms underlying the association between internal migration and dietary habits, an exploratory causal mediation analysis was conducted within a counterfactual framework. Mealtime social disconnection was specified as the mediator, and dietary habits score (without the “meal companionship” questions) was modeled as a continuous outcome. The average causal mediation effect (ACME), average direct effect (ADE), and total effect were estimated, along with the proportion mediated [32]. Confidence intervals for mediation effects were obtained using nonparametric bootstrap resampling, acknowledging the assumptions of no unmeasured confounding of the exposure–outcome, exposure–mediator, mediator–outcome relationships, and correct temporal ordering of variables. Due to these strong assumptions, the mediation results should be interpreted cautiously.

Sensitivity to potential unmeasured confounding was further evaluated using E-values for statistically significant associations [33]. E-values were calculated for both the point estimates and the lower bounds of the confidence intervals to quantify the minimum strength of association an unmeasured confounder would need to have with both the exposure and poor dietary habits, conditional on the measured covariates, to fully explain away the observed effects. Additional sensitivity analyses were performed. We calculated Odds Ratios (ORs) and 95% CI using multivariate logistic regressions instead of PRs to evaluate model output consistency. Moreover, we calculated ORs and PRs of poor dietary habits using the total DHQ-P score, including the “meal companionship” questions. This analysis will evaluate the robustness of our results to potential exposure-outcome overlap. Finally, we explore the effect of students’ university of origin as a potential confounder in our models.

All statistical tests were two-sided, with statistical significance defined as *p* < 0.05. Analyses were conducted using R (version 4.3.2), including standard packages for regression, mediation analysis, and sensitivity analyses.

## 3. Results

### 3.1. Sample Characteristics

Of the 1025 students enrolled at the index university, a target sample of 217 participants was defined. A total of 230 students were contacted and completed the survey. After exclusions due to international student status (n = 5) and incongruent responses to migration-related questions (n = 2), the final analytical sample included 223 medical students (Supplementary Figure S2). The study included 223 medical students, with a median age of their early twenties. Overall, poor dietary habits were prevalent, affecting approximately 47% of participants. Mealtime social disconnection was common: 35.8% of students reported eating all meals alone, and nearly half reported eating at least one daily meal without companionship. Alcohol consumption was also highly prevalent, with 67% of students reporting alcohol use more than once per month.

Socioeconomic characteristics indicated that most students relied on university scholarships (approximately 75% overall), and income levels varied across the sample, reflecting socioeconomic heterogeneity among medical students. Internal migrants represented 44% of the study population, allowing for meaningful comparisons between migrant and non-migrant students with respect to dietary habits and social and behavioral factors (Table 1).

Migrant students exhibited lower dietary habits than non-migrants, reflected in both a lower median dietary habits score and a higher prevalence of poor dietary habits. Migrants were also older and more likely to report receiving university scholarships compared with non-migrant students (Table 1).

The prevalence of alcohol consumption and mealtime social disconnection did not differ significantly by migration status.

### 3.2. Dietary Habits in Medical Students

**Figure 1:**
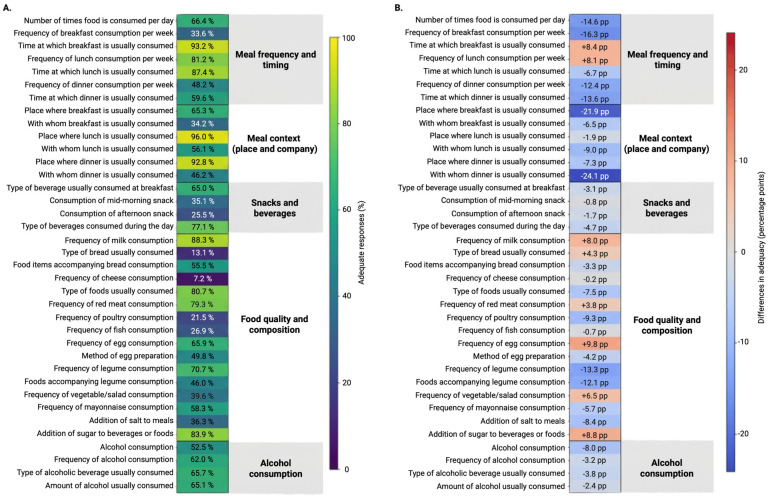
Dietary habits adequacy across DHQ-P items and differences by internal migration status among Peruvian medical students. (**A**) Percentage of adequate DHQ-P scores per item among all medical students surveyed; (**B**) Differences in DHQ-P scores per item of medical students with recent internal migration compared to their non-migrant peers.

Overall dietary habits among the 223 medical students showed substantial heterogeneity across dietary behaviors and domains (Figure 1A). The DHQ-P demonstrated acceptable internal consistency in this sample (Cronbach’s α = 0.74). The median DHQ-P score was approximately 30, consistent with prior studies using this instrument. Adequate responses were most frequently observed for meal timing and location, with high adequacy for the usual time of breakfast (93.2%), lunch (87.4%), and dinner (59.6%), as well as for the place where lunch (96.0%) and dinner (92.8%) were usually consumed. In contrast, meal regularity and food composition indicators revealed marked gaps, particularly for the frequency of breakfast consumption per week (33.6%), dinner consumption per week (48.2%), and consumption of mid-morning (35.1%) and afternoon snacks (25.5%).

Pronounced inadequacies were observed in several food quality and composition items, including low adequacy for the type of bread usually consumed (13.1%), frequency of cheese consumption (7.2%), frequency of poultry (21.5%) and fish consumption (26.9%), and vegetable/salad consumption (39.6%). In contrast, higher adequacy was noted for milk consumption (88.3%), red meat consumption (79.3%), legume consumption (70.7%), and the addition of sugar to foods or beverages (83.9%). Alcohol-related items showed intermediate adequacy levels, with 52.5% for alcohol consumption overall and approximately 62–66% for frequency, type, and amount of alcohol consumed.

When stratified by internal migration status, clear differences in dietary habits patterns emerged. Compared with non-migrant students, migrant students exhibited lower adequacy across multiple domains, particularly in meal frequency and timing and meal context (place and company). The largest absolute differences favored non-migrants for the frequency of breakfast and dinner consumption, shared meals (especially breakfast and dinner companionship), and several indicators of food quality, including fish and poultry consumption. Differences in alcohol-related items were more modest and less consistent between groups.

A delta heatmap summarizing the difference in adequacy between migrant and non-migrant students highlighted that most negative deltas (migrants minus non-migrants) clustered around social and structural aspects of eating, including meal regularity, shared meals, and selected food composition indicators (Figure 1B). These findings suggest that beyond individual food choices, contextual and social dimensions of eating play a central role in shaping dietary habits among medical students, particularly for those who migrate internally for education.

Panel A shows the proportion of students meeting adequacy criteria for each DHQ-P item, organized by dietary domain. Panel B displays the difference in adequacy (percentage points) between migrant and non-migrant students (migrants minus non-migrants) using a diverging color scale, where positive values indicate higher adequacy among migrants and negative values indicate lower adequacy. Domains include meal frequency and timing, meal context (place and company), snacks and beverages, food quality and composition, and alcohol consumption.

### 3.3. Associated Factors to Poor Dietary Habits

**Figure 2:**
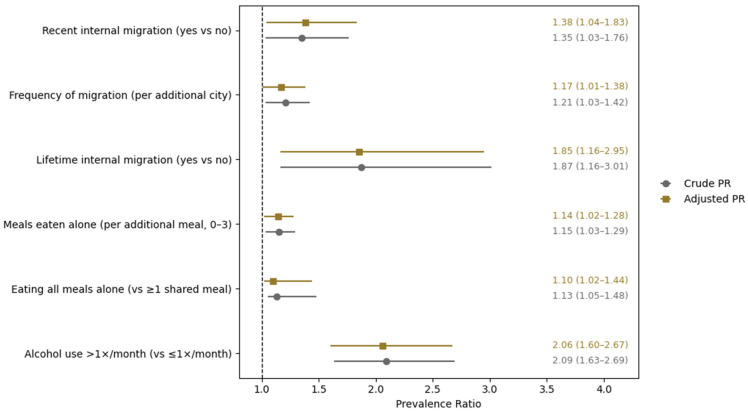
Factors associated with poor dietary habits among Peruvian medical students.

We tested separate multivariable analyses for the covariates of interest (social disconnection, internal migration and alcohol consumption) adjusted by age, semester, gender, and weekly income. All mealtime social disconnection, internal migration, and alcohol consumption were each independently associated with poor dietary habits (Figure 2). Full models are presented in Supplementary Results S1. Students who reported greater mealtime social disconnection had a substantially higher prevalence of poor dietary habits. When modeled as an ordinal exposure, each additional daily meal eaten alone was associated with a 14% increase in the prevalence of poor dietary habits (adjusted PR [aPR] = 1.14; 95% CI, 1.02–1.28). Consistently, students who reported eating all meals alone had a higher prevalence of poor dietary habits compared with those who shared at least one daily meal (aPR = 1.10; 95% CI, 1.02–1.44) (Figure 2).

A clear dose–response relationship was observed between the number of meals eaten alone and poor dietary habits. The progressive increase in prevalence across higher levels of solitary eating, evident in both crude and adjusted models, supports a graded association rather than a threshold effect. This pattern strengthens the plausibility of mealtime social disconnection as a meaningful behavioral correlate of dietary habits among medical students (Figure 2).

Internal migration was also significantly associated with poor dietary habits. Recent migrant students had 38% higher prevalence of poor dietary habits compared with non-migrant students (aPR = 1.38; 95% CI, 1.04–1.83), independent of age, gender, medical school semester, and income. Moreover, lifetime internal migration was associated with poor dietary habits (aPR = 1.85; 95% CI, 1.16–2.95). Interestingly, each additional new migration process to a different city was associated with an 17% increase in the prevalence of poor dietary habits (aPR = 1.17; 95% CI, 1.01–1.38). Similarly, to mealtime social disconnection, internal migration has a dose–response relationship with poor dietary habits.

Finally, alcohol consumption more than once per month was associated with poorer dietary habits (aPR = 2.06; 95% CI, 1.60–2.67). For all exposures, crude and adjusted estimates were nearly identical, indicating minimal confounding by the variables included in the multivariable models.

No statistically significant effect modification was observed between internal migration and either mealtime social disconnection (*p* = 0.09) or alcohol use (*p* = 0.99), suggesting that these factors contribute independently to poor dietary habits.

Forest plot showing crude and adjusted prevalence ratios (PRs) with 95% confidence intervals for poor dietary habits (modified DHQ-P score < 30). Associations are presented for internal migration characteristics (recent migration, lifetime migration, and frequency of migration), mealtime social disconnection, and alcohol use. Adjusted models were controlled for age, sex, academic semester, and income. Numeric PRs with 95% confidence intervals are displayed to the right of the plot. The vertical dashed line indicates the null value (PR = 1).

### 3.4. Mediation Analysis

**Figure 3:**
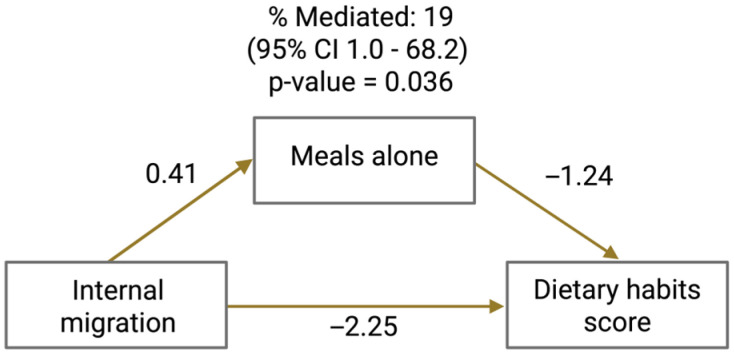
Mediation analysis of the association between internal migration and dietary habits through mealtime social disconnection.

Mediation analysis was conducted to examine whether mealtime social disconnection, operationalized as the number of meals eaten alone, mediated the association between recent internal migration and dietary habits score (Figure 3). Recent internal migration was significantly associated with a higher number of meals eaten alone, which in turn was associated with lower dietary habits scores.

The average causal mediation effect (ACME) was statistically significant (estimate = −0.52; 95% CI, −1.14 to −0.03; *p* = 0.030), indicating that mealtime social disconnection accounted for a meaningful indirect effect of recent internal migration on dietary habits (Table 2). The average direct effect (ADE) of internal migration on dietary habits score remained significant after accounting for the mediator (estimate = −2.25; 95% CI, −4.15 to −0.23; *p* = 0.024), supporting the presence of partial mediation. The total effect of internal migration on dietary habits score was also significant (estimate = −2.76; 95% CI, −4.64 to −0.71; *p* = 0.006).

Overall, mealtime social disconnection mediated approximately 19% of the association between recent internal migration and dietary habits (95% CI, 1–68%; *p* = 0.036). These findings suggest that reduced social exposure during meals is consistent with a possible behavioral pathway linking internal migration to poorer dietary habits, while also indicating that additional mechanisms beyond mealtime social disconnection contribute to the observed association.

The diagram illustrates the estimated direct and indirect pathways linking internal migration to dietary habits score, with mealtime social disconnection (meals eaten alone) as the mediator. Path coefficients represent regression estimates from the mediation models. The percentage mediated indicates the proportion of the total effect of internal migration on dietary habits explained by mealtime social disconnection.

### 3.5. Sensitivity Analysis

#### 3.5.1. Alternative Models

Sensitivity analyses using ORs with the modified DHQ-P score (Supplementary Results S2) and using ORs and PRs with the total DHQ-P score yielded estimates that were consistent in direction and magnitude with the primary prevalence ratio analyses (Supplementary Results S3 and S4). Additionally, we performed a sensitivity analysis by adding an extra potential confounder, the students’ university. The inclusion of this variable did not modify the association metrics (PR change from 0.01 to 0.5%); therefore, we removed the term and maintained the most parsimonious models.

#### 3.5.2. E-Value

Sensitivity analyses using E-values were conducted to assess the robustness of the observed associations to potential unmeasured confounding (Table 3). The E-values for the point estimates ranged from 2.04 to 3.94, indicating that an unmeasured confounder would need to be associated with both the exposure and poor dietary habits by risk ratios of this magnitude—above and beyond the measured covariates—to fully explain away the observed associations. These findings suggest that the observed associations are moderately robust to unmeasured confounding, as an unmeasured factor would need to exert a relatively strong and independent association with both the exposures and poor dietary habits to fully attenuate the results.

## 4. Discussion

### 4.1. Summary of Findings

In this study of Peruvian medical students, poor dietary habits were common and closely linked to structural, social, and behavioral factors that extend beyond individual food choices. Internal migration emerged as a central associated factor of poor dietary habits, with evidence suggesting that both recent and cumulative migration experiences contribute to dietary vulnerability. These associations followed a clear gradient, indicating that repeated or sustained exposure to migration-related disruptions may progressively undermine dietary behaviors.

Our findings further indicate that the social context of eating plays a key integrative role in this relationship. Mealtime social disconnection was strongly associated with poor dietary behaviors and partially mediated the effect of internal migration, suggesting that migration may influence dietary habits in part by disrupting shared eating routines and social supports. Importantly, this mediation was observed even in the absence of large differences in the prevalence of solitary eating, underscoring the relevance of socially patterned behaviors as meaningful pathways linking broader life transitions to health-related habits.

Alcohol consumption was also independently associated with poor dietary habits, reinforcing the notion that dietary behaviors cluster with other lifestyle risks during medical training. Taken together, these findings highlight poor dietary habits among medical students as a multifactorial phenomenon shaped by migration-related instability, erosion of social eating practices, and co-occurring risk behaviors. Addressing these interconnected dimensions may be essential for improving nutritional health in future physicians, particularly in middle-income settings characterized by high internal mobility.

### 4.2. Comparison with Previous Studies

Our findings are consistent with prior research documenting suboptimal dietary patterns among medical students across diverse regions. Studies from Europe, Asia, and Latin America have reported low adherence to dietary guidelines, frequent meal skipping, and high consumption of ultra-processed foods among medical trainees [34,35,36,37,38]. Similar to our results, these studies suggest that dietary habits deteriorate during medical training, often coinciding with increased academic demands and lifestyle disruption.

However, most previous studies have focused primarily on individual-level factors, such as stress, sleep deprivation, or time constraints, without adequately addressing social and contextual associated factors. Our study extends the literature by demonstrating that social eating patterns and migration-related factors are strongly associated with dietary habits, even after adjustment for demographic and socioeconomic variables.

### 4.3. Role of Internal Migration as a Cultural Challenge for Diet Adaptation

Internal migration for education represents a major life transition that can profoundly reshape dietary behaviors. Migrants often face disruption of traditional food practices, altered food availability, and reduced access to familiar meals, particularly in countries with strong regional culinary identities such as Peru. Previous research on dietary acculturation has shown that migration—both international and internal—can lead to increased consumption of processed foods and reduced dietary diversity, especially during early adaptation phases [13,39].

In the context of medical education, migration may compound these challenges by intersecting with academic stress and time scarcity. Our findings suggest that internal migration is not merely a geographic transition but a cultural and behavioral challenge that may adversely affect dietary habits among medical students.

A novel contribution of this study is the identification of mealtime social disconnection as a mediating pathway linking internal migration to poor dietary habits. Although migrants and non-migrants reported similar prevalence of eating alone, mediation analysis demonstrated that eating alone explained a meaningful proportion of the migration–dietary habits association. This finding underscores that small shifts in social routines, when combined with strong associations with dietary outcomes, can yield substantial indirect effects.

Migration often entails separation from family networks and established social structures, which may reduce opportunities for shared meals. Even when migrants establish new peer relationships, academic pressures may limit routine social eating. Our findings align with sociological models suggesting that daily social practices, rather than social network size alone, play a critical role in shaping health behaviors [40].

Beyond mealtime social disconnection, several additional pathways may help explain how internal migration shapes dietary habits among Peruvian medical students. Internal migration often entails exposure to new, more obesogenic food environments, where fast food and ultra-processed products become more available and affordable than fresh, traditional foods, potentially driving poorer diet quality [41]. Migrants also face increased stress, depressive symptoms, and loneliness, which are linked to unhealthy lifestyle clusters including irregular meals and energy-dense “comfort” eating [42]. Acculturation and cultural adaptation of diet—progressive replacement of traditional dishes by convenience foods—further contribute to worsening nutritional profiles [43]. Finally, economic strain and food insecurity after migration can push students toward cheaper, less diverse, and less healthy foods, reinforcing migration-related dietary risk [44].

### 4.4. The Role of Social Disconnection in Dietary Habits

Eating is a fundamental social behavior, and shared meals have been associated with better dietary quality, regular meal timing, and healthier food choices across age groups [45,46]. Conversely, eating alone has been linked to lower intake of fruits and vegetables, higher consumption of convenience foods, and poorer overall dietary habits [47,48].

Our finding of a clear dose–response relationship between the number of meals eaten alone and poor dietary habits strengthens the evidence for a graded association rather than a simple threshold effect. Importantly, this association persisted after adjustment for demographic and academic factors, highlighting mealtime social disconnection as a potentially modifiable behavioral target within medical training environments.

Beyond its nutritional implications, mealtime social disconnection may also reflect or contribute to broader experiences of social isolation and loneliness. Loneliness has emerged as a major public health concern, with growing evidence linking it to adverse mental health outcomes, including depression, anxiety, psychological distress, and burnout [49,50]—conditions that are highly prevalent among medical students and trainees [4]. Importantly, loneliness is not merely the absence of social contact but a subjective experience of insufficient social connection, which may be exacerbated by disrupted routines, academic pressure, and relocation away from established social networks.

Accumulating evidence suggests that loneliness and social isolation are also associated with clusters of health-risk behaviors, including unhealthy eating patterns, physical inactivity, sleep disturbances, and increased substance use [51,52]. In this context, eating alone may function both as a marker and a mechanism of social disconnection, reinforcing maladaptive coping strategies such as alcohol use or irregular eating in response to stress. Experimental and longitudinal studies further indicate that social isolation can alter reward processing, stress reactivity, and self-regulation, potentially increasing vulnerability to poor dietary choices and other risk behaviors during periods of high cognitive and emotional demand [53,54].

These findings suggest that the observed association between mealtime social disconnection and poor dietary habits likely reflects more than an isolated eating behavior. Rather, it may represent a broader psychosocial process in which disrupted social integration contributes to unhealthy lifestyle patterns and increased vulnerability to mental health problems. Furthermore, other affective and interpersonal processes, such as moral emotions like guilt, shame, and disgust, can influence eating-related behaviors and contribute to social withdrawal [55]. Given that mental health conditions and substance use disorders contribute highly to the burden of disease in Peru [56], interventions aimed at improving dietary habits among medical students are a priority. They may therefore benefit from addressing social connectedness and shared eating opportunities as part of a comprehensive approach to health promotion and well-being.

### 4.5. Alcohol Use and Dietary Habits

Alcohol consumption emerged as an independent correlate of poor dietary habits in this population. Alcohol use has been consistently associated with unhealthy dietary patterns, including higher intake of energy-dense foods, irregular meal timing, and increased consumption of ultra-processed products [57,58]. Among university students, alcohol use frequently clusters with other risk behaviors, such as late-night eating and meal skipping, which may further compromise dietary habits.

In our study, alcohol use was not an effect modifier of the migration—diet relationship, suggesting that alcohol represents a parallel behavioral risk factor rather than a mediating mechanism. Nonetheless, its high prevalence underscores the importance of addressing alcohol consumption within comprehensive health promotion strategies for medical students.

The fact that the frequency of alcohol consumption was positioned as an independent correlate of poor dietary habits in our study but not as a mediator of migration effects suggests that alcohol may influence eating patterns through several mechanisms that operate in parallel to migration-related processes. For example, alcohol consumption is often embedded in late-night socializing and night-eating patterns, which are associated with poorer eating habits, including skipping breakfast and higher fat intake among university students [59]. Furthermore, alcohol can stimulate appetite and increase total energy and food intake, partly through effects on satiety and reward pathways, which may promote energy-dense “comfort” eating [60]. These additional factors, which are relevant in the context of medical students, could be further scrutinized in future studies.

### 4.6. Public Health Applications and Future Directions

These findings have important implications for medical education and public health. Interventions aimed at improving dietary habits among medical students should move beyond individual nutrition education and consider social and structural strategies, such as promoting shared meals, improving access to healthy food options on campus, and supporting migrant students during transition periods.

Medical schools and teaching hospitals could implement peer-based meal programs, protected lunch times, weight and metabolic management programs [61], or culturally inclusive dining initiatives to reduce social disconnection. These recommendations are only hypothesis-generating statements that would benefit from evaluation using randomized control trials or longitudinal observational designs. Future studies are needed to assess temporal relationships and to evaluate whether interventions targeting mealtime social contexts can improve dietary habits and downstream health outcomes among medical trainees.

### 4.7. Limitations and Strengths

Several limitations must be acknowledged. First, the cross-sectional nature of our study precludes causal inference and introduces potential reverse causality; for example, students with poorer dietary routines or irregular schedules may be more likely to eat alone, rather than social isolation driving dietary changes. Although E-value analyses suggested moderate robustness of the findings. Second, all dietary, alcohol consumption, and migration data were collected via self-report, which is susceptible to social desirability bias, recall inaccuracies, or underreporting—particularly for sensitive behaviors like alcohol intake. While we used validated instruments (e.g., food frequency questionnaire) and assured anonymity to mitigate this, objective measures (e.g., dietary biomarkers or alcohol assays) would strengthen future work. Mealtime social disconnection was assessed using behavioral proxies rather than validated loneliness scales, capturing objective social exposure rather than subjective social experience. Other validated scales (e.g., Alcohol Use Disorders Identification Test-Concise and binge frequency and quantity) should be considered in future studies to improve the detail of diet behavior assessment in medical students. Similarly, we did not include specific variables about the migration process and context, such as time since migration, rural-to-urban vs. urban-to-urban, and living arrangement (with family vs. alone). Additionally, the study was conducted mostly at a single medical school, which may limit generalizability. Third, our snowball sampling strategy, while effective for reaching hidden populations like internal migrants, introduces selection bias by relying on peer networks; this may overrepresent students with denser social ties or specific dietary clusters, limiting generalizability. These biases are inherent to non-probability venue-based recruitment in campus settings and underscore the need for probability-based studies to confirm our findings. We recognized that our analyses are exploratory and require future replication and validation.

Strengths include the use of a validated dietary habits score, the examination of understudied social determinants, the evaluation of dose–response relationships, and the application of mediation and sensitivity analyses. To our knowledge, this is one of the first studies to examine internal migration, social eating patterns, and dietary habits among medical students in a middle-income country.

## 5. Conclusions

Poor dietary habits are highly prevalent among Peruvian medical students and are independently associated with internal migration, mealtime social disconnection, and alcohol consumption. Mealtime social disconnection partially mediated the relationship between migration and dietary habits, highlighting the importance of social routines in shaping dietary behaviors. Due to the cross-sectional nature of this study, causality cannot be inferred, and further longitudinal studies are required to understand these dynamics. Nevertheless, addressing social and contextual factors associated with diet may offer novel opportunities to improve the health and well-being of future physicians.

## Figures and Tables

**Table 1:** Characteristics of the participants.

Variables	Non-Migrant N = 125 ^1^	Migrant N = 98 ^1^	*p*-Value ^2^
**Dietary habits score**	33 (28, 36)	28 (24, 33)	<0.001
**Poor dietary habits**	47 (38%)	57 (58%)	0.002
**Age**	22.0 (20.0, 24.0)	24.0 (22.0, 26.0)	<0.001
**Gender**	49 (40%)	24 (24%)	0.016
**Missing**	2	0	
**Weekly income**			0.067
More than $148.2	28 (22%)	13 (13%)	
Less than $44.5	60 (48%)	43 (44%)	
Between $44.5 to $148.2	37 (30%)	42 (43%)	
**University**			>0.9
**UCV**	107 (86%)	86 (88%)	
**UNAP**	3 (2.4%)	2 (2%)	
**UNSA**	3 (2.4%)	1 (1%)	
**UPAO**	5 (4.0%)	3 (3.1%)	
**UPR**	4 (3.2%)	4 (4.1%)	
**Other**	3 (2.4%)	2 (2.0%)	
**University scholarships**	87 (70%)	80 (82%)	0.040
**Alcohol intake frequency**			0.4
≤1 time per month	87 (70%)	63 (64%)	
>1 time per month	38 (30%)	35 (36%)	
**N° of daily meals alone**			0.083
0	39 (31%)	20 (20%)	
1	28 (23%)	16 (16%)	
2	19 (15%)	20 (20%)	
3	38 (31%)	42 (43%)	
**Missing**	1	0	
**All meals alone**	38 (31%)	42 (43%)	0.060
**Missing**	1	0	
**Lifetime migration**			<0.001
Never	45 (36%)	0 (0%)	
Once	57 (46%)	67 (68%)	
Twice or more	23 (18%)	31 (32%)	

Footnotes: Non-migrant and migrant status were defined as having migrated for the purpose of studying or continuing education. ^1^ Median (Q1, Q3); n (%), ^2^ Wilcoxon rank sum test; Pearson’s Chi-squared test.

**Table 2:** Mediation analysis effects.

Type of Effects	Estimate	95% CI Lower	95% CI Upper	*p*-Value
ACME	−0.52	−1.14	−0.03	0.030 *
ADE	−2.25	−4.15	−0.23	0.024 *
Total Effect	2.76	−4.64	−0.71	0.006 **

Footnotes: ACME = Average Causal Mediation Effect, representing the indirect effect of internal migration on dietary habits through mealtime social disconnection. ADE = Average Direct Effect, representing the effect of internal migration on dietary habits not mediated by mealtime social disconnection. Total Effect = Sum of the direct and indirect effects. Estimates represent changes in the continuous dietary habits score (DHQ-P), with negative values indicating poorer dietary habits score. Confidence intervals were obtained using nonparametric bootstrapping. * *p* < 0.05; ** *p* < 0.01.

**Table 3:** E-values estimations.

Exposure	Model Specification	E-Value for Point Estimate	E-Value for Confidence Interval
Mealtime social disconnection	Number of meals eaten alone (0–3)	1.38	1.11
	Eating all meals alone vs. ≥1 shared meal	1.34	1.11
Internal migration	Recent Migrant vs. non-migrant	1.63	1.16
	Lifetime Migrant vs. non-migrant	2.06	1.37
	Frequency of migration (1–4 places)	1.38	1.08
Alcohol Use	>1 time per month vs. ≤1 time per month	2.23	1.84

Footnotes: E-values quantify the minimum strength of association that an unmeasured confounder would need to have with both the exposure and the outcome, conditional on the measured covariates, to fully explain away the observed association. The E-value for the point estimate represents the minimum confounding strength required to reduce the observed association to the null. The E-value for the confidence interval corresponds to the minimum confounding strength required to shift the lower bound of the 95% confidence interval to include the null value. Larger E-values indicate greater robustness of the observed association to potential unmeasured confounding. E-values were calculated based on adjusted prevalence ratios from multivariable logistic regression models.

## Data Availability

The data presented in this article are part of a thesis to obtain the Medical Doctor Degree conducted at the Cesar Vallejo University (JH-B). The data presented in this study are available on request from the corresponding author. The data are not publicly available due to privacy restrictions.

## Supplementary Materials

The following supporting information can be downloaded at https://www.mdpi.com/article/10.3390/healthcare14040433/s1, Figure S1: Conceptual directed acyclic graph (DAG) of internal migration and poor dietary habits; Figure S2: Participant recruitment and inclusion flowchart; Methods S1: Dietary History Questionnaire—Peruvian version (DHQ-P); Methods S2: Description of the Questionnaire Domains and included items; Results S1: PRs of each covariate of the main regression models applied (excluding eating alone questions); Results S2: ORs of each covariate of the main regression models applied (excluding eating alone questions); Results S3: PRs of each covariate of the regression models applied including “eating alone” questions; Results S4. ORs of each covariate of the regression models applied including “eating alone” questions.
