# Supplementary material for: Internal Migration, Mealtime Social Disconnection, and Alcohol Use Are Linked to Poor Dietary Habits Among Peruvian Medical Students
Source: Healthcare (Basel). 2026 Feb 9;14(4):433. doi: 10.3390/healthcare14040433 (PMC12941228; doi:10.3390/healthcare14040433)
Supplement: Supplementary file 1 [file healthcare-14-00433-s001.zip › healthcare-4107245-supplementary.pdf]

This is the Supplementary Material from

Internal Migration, Mealtime Social Disconnection, and Alcohol Use are Linked to Poor Dietary Habits Among Peruvian Medical Students

Alba Navarro-Flores 1,2,†,*, Josue Humpire-Belizario 3,†, Frances Condori 4,5 and Kevin Pacheco-Barrios 5,6,*

1 Institute of Psychiatric Phenomics and Genomics (IPPG), LMU University Hospital, LMU Munich, Munich, Germany; 2 International Max Planck Research School for Translational Psychiatry (IMPRS-TP), Munich, Germany; 3 Escuela de Medicina, Universidad Cesar Vallejo, Trujillo 13001, Peru; 4 IvyBridge Program, Philltec, Lima, Peru; 5 Neuromodulation Center and Center for Clinical Research Learning, Spaulding Rehabilitation Hospital and Massachusetts General Hospital, Harvard Medical School, Boston, Massachusetts; 6 Unidad de Investigación para la Generación y Síntesis de Evidencias en Salud, Vicerrectorado de Investigación, Universidad San Ignacio de Loyola, Lima, Peru. † Equally contributing authors. *Correspondence

Content

Supplementary Figure S1. Conceptual directed acyclic graph (DAG) of internal migration and poor dietary habits. 2

Supplementary Figure S2. Participant recruitment and inclusion flowchart........ 3

Supplementary Methods S1. Dietary History Questionnaire - Peruvian version (DHQ-P)........ 3

Supplementary Methods S2. Description of the Questionnaire Domains and included items 7

Supplementary Results S1. PRs of each covariate of the main regression models applied (excluding eating alone questions)........ 9

Supplementary Results S2. ORs of each covariate of the main regression models applied (excluding eating alone questions)........ 9

Supplementary Results S3. PRs of each covariate of the regression models applied including “eating alone” questions 10

Supplementary Results S4. ORs of each covariate of the regression models applied including “eating alone” questions 10

Supplementary Figure S1. Conceptual directed acyclic graph (DAG) of internal migration and poor dietary habits.

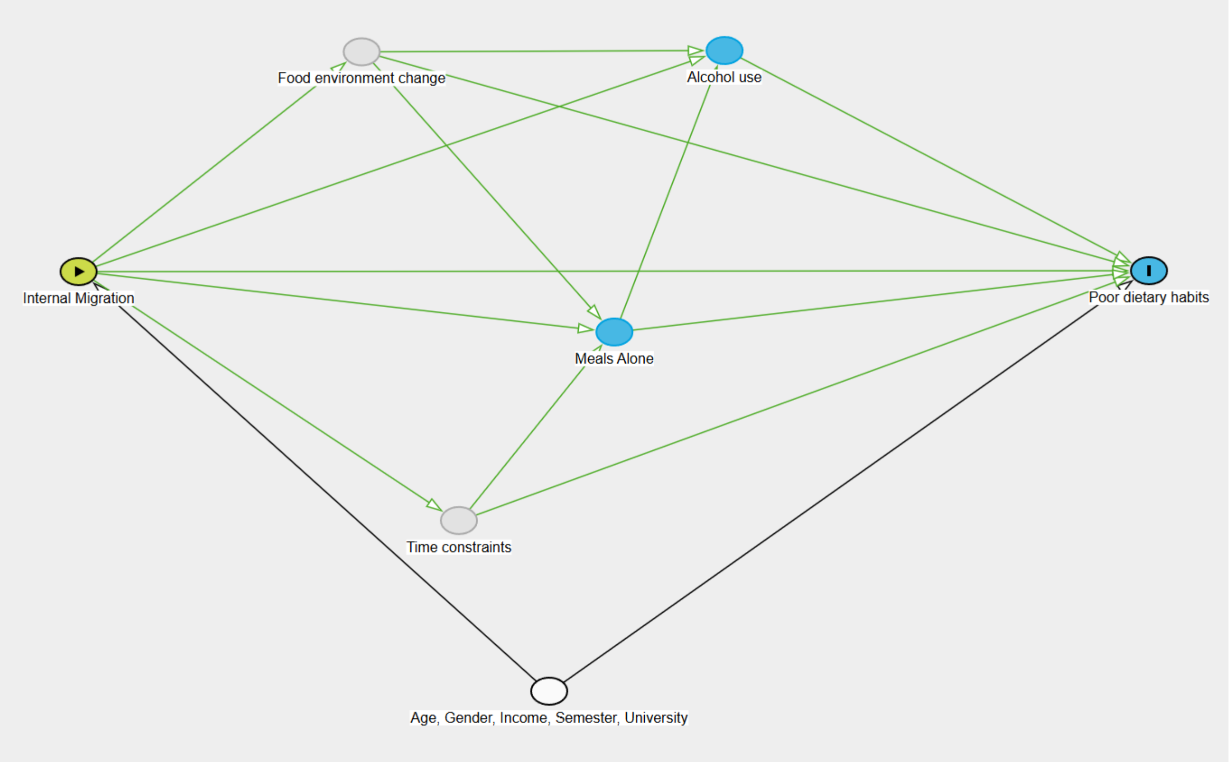

The DAG illustrates the hypothesized relationships between internal migration and poor dietary habits among medical students. Internal migration is modeled as the primary exposure, with meals eaten alone, alcohol use, food environment changes, and time constraints conceptualized as potential mediators. Age, gender, income, medical school semester, and university are treated as confounders. Arrows indicate assumed directional relationships based on prior literature and theoretical considerations.

Supplementary Figure S2. Participant recruitment and inclusion flowchart

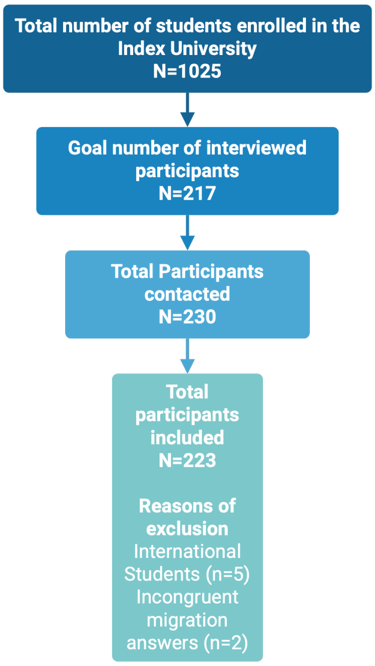

Flow diagram describing the recruitment, screening, and inclusion of medical students in the study. Of the 1,025 students enrolled at the index university, a target sample of 217 participants was defined. A total of 230 students were contacted and completed the survey. After exclusions due to international student status (n = 5) and incongruent responses to migration-related questions (n = 2), the final analytical sample included 223 medical students.

Supplementary Methods S1. Dietary History Questionnaire - Peruvian version (DHQ-P)

Item	Answers	Adequate habits options (score)
**Item 1: Meal Frequency**<br>**How many times a day do you consume food?**	A. Less than 3 times a day<br>B. 3 times a day<br>C. 4 times a day<br>D. 5 times a day<br>E. More than 5 times a day	Adequate habits (2 points):<br>B, C, D
**Item 2: Breakfast Frequency per Week**<br>**How many times a week do you have breakfast?**	A. Never<br>B. 1–2 times a week<br>C. 3–4 times a week<br>D. 5–6 times a week<br>E. Daily	Adequate habits (2 points):<br>E
**Item 3: Breakfast Time**<br>**At what time do you mainly have breakfast?**	A. 4:00–4:59 a.m.<br>B. 5:00–5:59 a.m.<br>C. 6:00–6:59 a.m.<br>D. 7:00–7:59 a.m.<br>E. 8:00–8:59 a.m.	Adequate habits (1 point):<br>C, D, E
**Item 4: Breakfast Location**<br>**Where do you have breakfast?**	A. At home<br>B. At university<br>C. At a restaurant<br>D. At kiosks<br>E. At street stalls	Adequate habits (1 point):<br>A, B, C

**Item 5: Breakfast Companionship**<br>**With whom do you have breakfast?**	A. With family<br>B. With friends<br>C. With study companions<br>D. With work colleagues<br>E. Alone	Adequate habits (1 point):<br>A, B, C, D
**Item 6: Breakfast Beverage**<br>**What beverage do you usually drink at breakfast?**	A. Milk and/or yogurt<br>B. Fruit juices<br>C. Oatmeal, quinoa, soy<br>D. Infusions and/or coffee<br>E. Others, specify:	Adequate habits (2 points):<br>A, C
**Item 6A: Milk Consumption Frequency**<br>**How often do you drink milk?**	A. Never<br>B. 1–2 times a month<br>C. 1–2 times a week<br>D. Every other day<br>E. Daily	Adequate habits (2 points):<br>D, E
**Item 7: Bread Consumption**<br>**Do you consume bread?**	A. Yes<br>B. No (go to question 8)	
**Item 7A: Bread Accompaniments**<br>**What do you usually pair bread with?**	A. Cheese and/or chicken<br>B. Avocado or olives<br>C. Processed meats<br>D. Butter and/or fried foods (fried egg, omelet)<br>E. Jam and/or *dulce de leche*	Adequate habits (2 points):<br>Question 7: B or Question 7A: A, B
**Item 7B: Bread Type**<br>**If you do consume bread, what type do you usually eat?**		Optional question, no score
**Item 8: Cheese Consumption Frequency**<br>**How often do you consume cheese?**	A. Never<br>B. 1–2 times a month<br>C. 1–2 times a week<br>D. Every other day<br>E. Daily	Adequate habits (2 points):<br>D, E
**Item 9: Mid-Morning Snack**<br>**What do you usually consume mid-morning?**	A. Fruits (whole or prepared)<br>B. Hamburger<br>C. Snacks and/or cookies<br>D. Ice cream<br>E. Nothing	Adequate habits (2 points):<br>A
**Item 10: Mid-Afternoon Snack**<br>**What do you usually consume mid-afternoon?**	A. Fruits (whole or prepared)<br>B. Hamburger<br>C. Snacks and/or cookies<br>D. Ice cream<br>E. Nothing	Adequate habits (2 points):<br>A
**Item 11: Daytime Beverages**<br>**What beverages do you usually consume during the day?**	A. Plain water<br>B. Soft drinks<br>C. Infusions<br>D. Sodas<br>E. Industrialized juices<br>F. Rehydrating drinks	Adequate habits (2 points):<br>A, B
**Item 12: Lunch Frequency per Week**<br>**How many times a week do you have lunch?**	A. Never<br>B. 1–2 times a week<br>C. 3–4 times a week<br>D. 5–6 times a week<br>E. Daily	Adequate habits (2 points):<br>E
**Item 13: Lunch Time**<br>**At what time do you mainly have lunch?**	A. 11:00–11:59 a.m.<br>B. 12:00–12:59 p.m.<br>C. 1:00–1:59 p.m.<br>D. 2:00–2:59 p.m.<br>E. After 3:00 p.m.	Adequate habits (1 point):<br>B, C, D

**Item 14: Lunch Location**<br>**Where do you have lunch?**	A. At home<br>B. At university<br>C. At a restaurant<br>D. At kiosks<br>E. At street stalls	Adequate habits (1 point):<br>A, B, C
**Item 15: Lunch Food Type**<br>**What do you usually consume at lunch?**	A. Creole food<br>B. Vegetarian food<br>C. Light food<br>D. Rotisserie chicken and/or pizzas<br>E. Junk food	Adequate habits (2 points):<br>A
**Item 16: Lunch Companionship**<br>**With whom do you have lunch?**	A. With family<br>B. With friends<br>C. With study companions<br>D. With work colleagues<br>E. Alone	Adequate habits (1 point):<br>A, B, C, D
**Item 17: Dinner Frequency per Week**<br>**How many times a week do you have dinner?**	A. 1–2 times a week<br>B. 3–4 times a week<br>C. 5–6 times a week<br>D. Daily	Adequate habits (2 points):<br>D
**Item 18: Dinner Time**<br>**At what time do you usually have dinner?**	A. 5:00–5:59 p.m.<br>B. 6:00–6:59 p.m.<br>C. 7:00–7:59 p.m.<br>D. 8:00–8:59 p.m.<br>E. After 9:00 p.m.	Adequate habits (1 point):<br>B, C, D
**Item 19: Dinner Location**<br>**Where do you have dinner?**	A. At home<br>B. At university<br>C. At a restaurant<br>D. At kiosks<br>E. At street stalls	Adequate habits (1 point):<br>A, B, C
**Item 20: Dinner Food Type**<br>**What do you usually consume at dinner?**	A. Creole food<br>B. Vegetarian food<br>C. Light food<br>D. Rotisserie chicken and/or pizzas<br>E. Junk food	Adequate habits (2 points):<br>A
**Item 21: Dinner Companionship**<br>**With whom do you have dinner?**	A. With family<br>B. With friends<br>C. With study companions<br>D. With work colleagues<br>E. Alone	Adequate habits (1 point):<br>A, B, C, D
**Item 22: Food Preparation Method**<br>**What type of preparation do you most often consume in your main meals during the week?**	A. Stewed<br>B. Boiled<br>C. Fried<br>D. Baked<br>E. Grilled	Adequate habits (2 points):<br>A, B, D, E
**Item 23: Beef or Pork Consumption**<br>**How often do you consume beef or pork?**	A. Never<br>B. 1–2 times a month<br>C. 1–2 times a week<br>D. Every other day<br>E. Daily	Adequate habits (2 points):<br>B, C
**Item 24: Chicken Consumption**<br>**How often do you consume chicken?**	A. Never<br>B. 1–2 times a month<br>C. 1–2 times a week<br>D. Every other day<br>E. Daily	Adequate habits (2 points):<br>C
**Item 25: Fish Consumption**<br>**How often do you consume fish?**	A. Never<br>B. 1–2 times a month<br>C. 1–2 times a week	Adequate habits (2 points):<br>C

	D. Every other day<br>E. Daily	
**Item 26: Egg Consumption**<br>**How often do you consume eggs?**	A. Never<br>B. 1–2 times a month<br>C. 1–2 times a week<br>D. Every other day<br>E. Daily	Adequate habits (2 points): C, D and Item 26A A, C, D
**Item 26A: Egg Preparation Method**<br>**In what type of preparation do you most often consume eggs?**	A. Boiled<br>B. Fried<br>C. Poached<br>D. Soft-boiled<br>E. In prepared dishes	
**Item 27: Legumes Consumption**<br>**How often do you consume legumes (beans, lentils, chickpeas)?**	A. Never (go to question 28)<br>B. 1–2 times a month<br>C. 1–2 times a week<br>D. Every other day<br>E. Daily	Adequate habits (2 points): C, D
**Item 27A: Beverage with Legumes**<br>**What beverage do you usually drink with your legume dish?**	A. Citrus drinks (lemonade, papaya or orange juice)<br>B. Infusions (tea, anise, chamomile)<br>C. Sodas<br>D. Water	Adequate habits (1 point): A
**Item 28: Vegetable Salad Consumption**<br>**How often do you consume vegetable salads?**	A. Never<br>B. 1–2 times a month<br>C. 1–2 times a week<br>D. Every other day<br>E. Daily	Adequate habits (2 points): D, E
**Item 29: Mayonnaise Consumption**<br>**How often do you consume mayonnaise with your meals?**	A. Never<br>B. 1–2 times a month<br>C. 1–2 times a week<br>D. Every other day<br>E. Daily	Adequate habits (2 points): A, B
**Item 30: Salt Addition to Meals**<br>**Do you usually add salt to prepared meals?**	A. Never<br>B. 1–2 times a month<br>C. 1–2 times a week<br>D. Every other day<br>E. Daily	Adequate habits (2 points): A
**Item 31: Sugar in Beverages**<br>**How many teaspoons of sugar do you add to a glass or cup?**	A. None<br>B. 1 teaspoon<br>C. 2 teaspoons<br>D. 3 teaspoons<br>E. More than 3 teaspoons	Adequate habits (2 points): A, B, C
**Domain: Alcohol consumption (1 item and 3 subitems, maximum score: 2 pts)**		
**Item 32: Alcoholic Beverage Consumption**<br>**Do you consume alcoholic beverages?**	A. Yes (go to question 32A)<br>B. No	Adequate habits (2 points): Question 32 is B<br>or<br>Question 32 is A and Questions 32A, 32B, 32C are adequate
**Item 32A: Alcoholic Beverage Frequency**<br>**How often do you consume alcoholic beverages?**	A. Less than 1 time a month<br>B. 1 time a month<br>C. 1–2 times a month<br>D. 1 time a week<br>E. More than 1 time a week	Adequate habits: A, B

**Item 32B: Type of Alcoholic Beverage**<br>**What type of alcoholic beverage do you most often consume?**	A. Pisco<br>B. Wine<br>C. Sangria<br>D. Beer<br>E. Others	Adequate habits: B
**Item 32C: Quantity of Alcoholic Beverage**<br>**What quantity do you usually drink of the alcoholic beverage?**	A. One glass<br>B. 1–2 glasses<br>C. 3–4 glasses<br>D. 5–6 glasses<br>E. More than 6 glasses	Adequate habits: A, B

Legend. The options considered as adequate are listed in the last column. Only one option is possible to be selected per question.

Supplementary Methods S2. Description of the Questionnaire Domains and included items

Domains	Items	Maximum Score
Number of main meals	Item 1 (2)	2 pts
Frequency of main meals per week	Item 2 (2)<br>Item 12 (2)<br>Item 17 (2)	6 pts
Time of eating	Item 3 (1)<br>Item 13 (1)<br>Item 18 (1)	3 pts
Place of eating	Item 4 (1)<br>Item 14 (1)<br>Item 19 (1)	3 pts
Companionship	Item 5 (1)<br>Item 16 (1)<br>Item 21 (1)	3 pts
Type of food	Item 15 (2)<br>Item 20 (2)	4 pts
Food preparation	Item 22 (2)	2 pts
Snacks	Item 9 (2)<br>Item 10 (2)	4 pts
Beverage consumption	Item 6 (2)<br>Item 11 (2)<br>Item 27A (1)<br>Item 32 (including 32A, 32B, and 32C) (2)	7 pts
Frequency of food component consumption	Item 6A (2)<br>Item 7 (including 7A and 7B) (2)<br>Item 8 (2)<br>Item 23 (2)<br>Item 24 (2)<br>Item 25 (2)<br>Item 26 (including 26A) (2)<br>Item 27 (2)<br>Item 28 (2)<br>Item 29 (2)<br>Item 30 (2)<br>Item 31 (2)	24 pts

10 Domains	32 Items (8 sub-items)	58 pts

Supplementary Results S1. PRs of each covariate of the main regression models applied (excluding eating alone questions)

Variables	Models of associated factors to dietary habits					
	Recent internal migration	Lifetime internal migration	Frequency of migration	Meals Eaten Alone	Eating all meals alone	Alcohol use
N	221	221	221	220	220	221
**Covariate of interest**	1.38 (1.04–1.83)	1.85 (1.16–2.95)	1.17 (1.01–1.38)	1.14 (1.02–1.28)	1.10 (1.02–1.44)	2.06 (1.60–2.67)
**Semester**	1.04 (0.98–1.09)	1.03 (0.98–1.09)	1.04 (0.98–1.09)	1.04 (0.99–1.10)	1.05 (0.99–1.10)	1.04 (0.99–1.09)
**Age**	0.87 (0.28–2.71)	1.13 (0.37–3.43)	0.92 (0.31–2.76)	1.14 (0.40–3.23)	1.10 (0.37–3.22)	0.95 (0.30–3.04)
**Sex**	1.41 (1.09–1.84)	1.36 (1.05–1.76)	1.33 (1.02–1.73)	1.37 (1.06–1.77)	1.35 (1.04–1.76)	1.16 (0.90–1.51)
**Income High vs Low**	0.97 (0.67–1.41)	0.98 (0.67–1.43)	1.00 (0.68–1.47)	1.09 (0.74–1.61)	1.08 (0.73–1.59)	1.03 (0.74–1.45)
**Income High vs Moderate**	0.89 (0.60–1.33)	0.89 (0.60–1.32)	0.95 (0.63–1.42)	1.02 (0.68–1.53)	1.01 (0.67–1.53)	1.01 (0.71–1.44)

Notes: PRs estimated via modified Poisson regression with log link and robust (sandwich) standard errors (HC0).

Supplementary Results S2. ORs of each covariate of the main regression models applied (excluding eating alone questions)

Variables	Models of associated factors to dietary habits					
	Recent internal migration	Lifetime internal migration	Frequency of migration	Meals Eaten Alone	Eating all meals alone	Alcohol use
**Covariate of interest**	1.93 (1.07–3.48)	2.97 (1.41–6.23)	1.43 (1.01–2.06)	1.31 (1.04–1.64)	1.11 (1.01–2.14)	5.57 (2.90–10.70)
**Semester**	1.07 (0.97–1.18)	1.06 (0.96–1.17)	1.07 (0.98–1.18)	1.08 (0.98–1.19)	1.09 (0.99–1.20)	1.09 (0.98–1.21)
**Age**	0.76 (0.09–6.37)	1.23 (0.14–10.51)	0.85 (0.10–7.01)	1.26 (0.15–10.32)	1.19 (0.15–9.68)	0.99 (0.10–9.51)
**Sex**	2.08 (1.15–3.77)	1.96 (1.08–3.54)	1.86 (1.04–3.33)	1.95 (1.08–3.52)	1.88 (1.05–3.36)	1.45 (0.78–2.72)
**Income High vs Low**	0.96 (0.44–2.06)	0.96 (0.44–2.09)	0.97 (0.45–2.11)	1.16 (0.53–2.55)	1.15 (0.53–2.50)	1.08 (0.48–2.46)
**Income High vs Moderate**	0.81 (0.36–1.81)	0.78 (0.35–1.77)	0.87 (0.39–1.94)	1.00 (0.44–2.27)	1.01 (0.45–2.26)	1.04 (0.44–2.44)

Supplementary Results S3. PRs of each covariate of the regression models applied including "eating alone" questions

Variables	Models of associated factors to dietary habits					
	Recent internal migration	Lifetime internal migration	Frequency of migration	Eating Alone	Eating all meals alone	Alcohol use
**Covariate of interest**	1.54 (1.13–2.08)	2.89 (1.52–5.49)	1.27 (1.08–1.49)	1.37 (1.21–1.56)	1.53 (1.17–2.01)	1.38 (1.04–1.83)
**Semester**	1.03 (0.97–1.09)	1.02 (0.97–1.08)	1.04 (0.98–1.09)	1.03 (0.98–1.09)	1.04 (0.99–1.10)	1.04 (0.98–1.10)
**Age**	0.93 (0.27–3.20)	1.33 (0.41–4.31)	0.97 (0.31–3.07)	1.39 (0.51–3.80)	1.30 (0.45–3.78)	1.16 (0.36–3.76)
**Sex**	1.15 (0.85–1.54)	1.09 (0.81–1.45)	1.06 (0.79–1.42)	1.12 (0.85–1.47)	1.08 (0.81–1.45)	1.01 (0.74–1.37)
**Income High vs Low**	1.39 (0.87–2.23)	1.39 (0.88–2.19)	1.43 (0.90–2.27)	1.66 (1.06–2.62)	1.66 (1.04–2.64)	1.49 (0.94–2.35)
**Income High vs Moderate**	1.18 (0.71–1.95)	1.16 (0.72–1.88)	1.27 (0.78–2.06)	1.44 (0.89–2.34)	1.46 (0.89–2.38)	1.33 (0.82–2.14)

Notes: PRs estimated via modified Poisson regression with log link and robust (sandwich) standard errors (HC0).

Supplementary Results S4. ORs of each covariate of the regression models applied including "eating alone" questions

Variables	Models of associated factors to dietary habits					
	Recent internal migration	Lifetime internal migration	Frequency of migration	Eating Alone	Eating all meals alone	Alcohol use
**Covariate of interest**	2.30 (1.29 - 4.17)	5.11 (2.30 - 12.60)	1.64 (1.14 - 2.39)	1.83 (1.44 - 2.36)	2.38 (1.34 - 4.31)	1.58 (1.05 - 2.42)
**Semester**	1.06 (0.96 - 1.17)	1.05 (0.95 - 1.16)	1.06 (0.97 - 1.18)	1.07 (0.96 - 1.18)	1.08 (0.98 - 1.19)	1.08 (0.98 - 1.19)
**Age**	0.91 (0.10 - 7.75)	1.80 (0.18 - 17.16)	1.02 (0.11 - 8.84)	2.03 (0.21 - 19.10)	1.69 (0.19 - 14.71)	1.32 (0.15 - 10.97)
**Sex**	1.31 (0.72 - 2.39)	1.20 (0.66 - 2.19)	1.13 (0.63 - 2.03)	1.24 (0.67 - 2.30)	1.14 (0.63 - 2.07)	1.01 (0.56 - 1.83)
**Income High vs Low**	1.85 (0.85 - 4.15)	1.91 (0.86 - 4.39)	1.91 (0.87 - 4.32)	2.72 (1.17 - 6.67)	2.58 (1.15 - 6.06)	2.07 (0.95 - 4.65)
**Income High vs Moderate**	1.34 (0.58 - 3.11)	1.30 (0.56 - 3.07)	1.48 (0.65 - 3.44)	1.96 (0.82 - 4.88)	1.94 (0.84 - 4.67)	1.65 (0.73 - 3.82)
